# Trends and challenges of fruit by-products utilization: insights into safety, sensory, and benefits of the use for the development of innovative healthy food: a review

**DOI:** 10.1186/s40643-023-00722-8

**Published:** 2024-01-18

**Authors:** Md. Mehedi Hasan, Md. Rakibul Islam, Ahmed Redwan Haque, Md. Raihan Kabir, Khursheda Jahan Khushe, S. M. Kamrul Hasan

**Affiliations:** 1 Department of Food Processing and Preservation, Hajee Mohammad Danesh Science and Technology University (HSTU), Dinajpur, 5200 Bangladesh; 2 Department of Food Science and Nutrition, Hajee Mohammad Danesh Science and Technology University (HSTU), Dinajpur, 5200 Bangladesh

**Keywords:** Fruit by-products, Health benefits, Healthy foods, Safety, Sensory

## Abstract

A significant portion of the human diet is comprised of fruits, which are consumed globally either raw or after being processed. A huge amount of waste and by-products such as skins, seeds, cores, rags, rinds, pomace, etc. are being generated in our homes and agro-processing industries every day. According to previous statistics, nearly half of the fruits are lost or discarded during the entire processing chain. The concern arises when those wastes and by-products damage the environment and simultaneously cause economic losses. There is a lot of potential in these by-products for reuse in a variety of applications, including the isolation of valuable bioactive ingredients and their application in developing healthy and functional foods. The development of novel techniques for the transformation of these materials into marketable commodities may offer a workable solution to this waste issue while also promoting sustainable economic growth from the bio-economic viewpoint. This approach can manage waste as well as add value to enterprises. The goal of this study is twofold based on this scenario. The first is to present a brief overview of the most significant bioactive substances found in those by-products. The second is to review the current status of their valorization including the trends and techniques, safety assessments, sensory attributes, and challenges. Moreover, specific attention is drawn to the future perspective, and some solutions are discussed in this report.

## Introduction

The Food and Agriculture Organization reports that approximately 14% of the world's food, worth $400 billion every year, is wasted before it reaches stores (Jenkins et al. 2016). Furthermore, the food that is lost or wasted every year could provide enough food for an average of 1.26 billion people. Fruits are an important diet for human health and it produces a lot of waste like other foods from domestic use and food-producing industries. During processing, almost half of each fruit is discarded as waste or by-products by the industries, which include peels, seeds, rinds, husks, rags, roots, pomace, etc. (Mahato et al. 2019). These by-products contain considerable quantities of bioactive substances that can damage the environment and the ecosystem due to their high biological oxygen demands. Over the past few decades, numerous research studies have improved our understanding of the use of fruit by-products and altered our perception of the positive impact of those by-products on human health (Cheok et al. 2018).

**Fig. 1:**
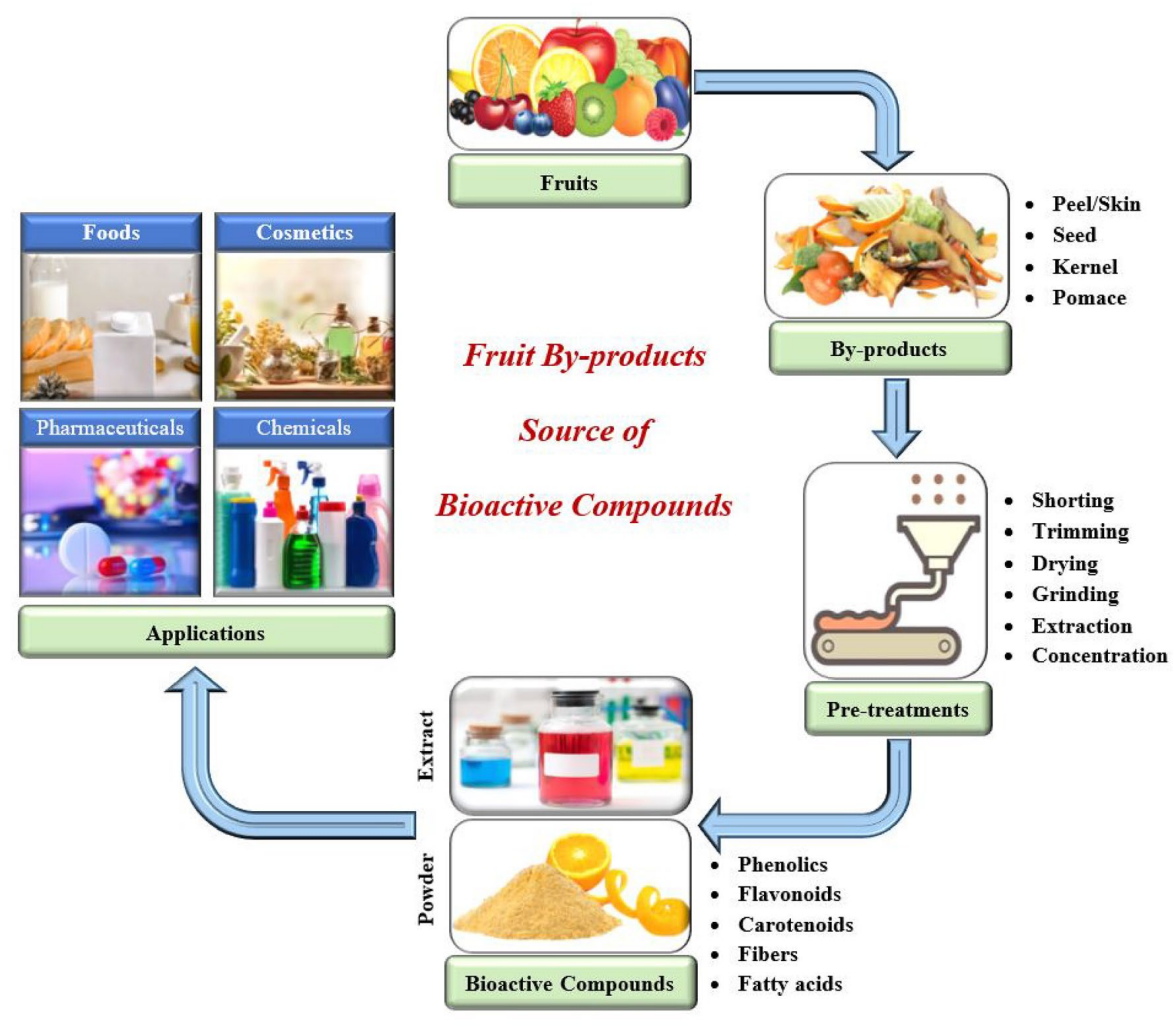
A conceptual example of extraction, isolation and application of bioactive compounds from FBPs

Fruit by-products (FBPs) are a great source of valuable bioactive substances like phenolic acids, carotenoids, flavonoids, dietary fibers, and more. Phytochemicals, phytosterols, and essential oils can be found in large quantities in fruit seeds and skins. Similarly, pectin, valuable fibers, and minerals may be found in the peel, rinds, and pomace (de Albuquerque et al. 2019; Islam et al. 2023a, b). These bioactive substances can be recovered from the by-products using a variety of technologies, and they can then be used to create a range of valorized products, such as dietary supplements or functional foods (Pathak et al. 2016; Kabir et al. 2021; Panza et al. 2021). For instance, the application of biotransformation processes using enzymes or microorganisms can convert FBPs into bioactive ingredients such as antioxidants, dietary fibers, protein, and essential oils (Sadh et al. 2018). These transformed products can find applications in the food, pharmaceutical, and cosmetic industries. Figure 1 represents the transformation of fruit by-products into valuable ingredients for foods, pharmaceuticals, and cosmetics. In this way, food loss or waste can be minimized and the risk to the environment can be mitigated.

The extraction and utilization techniques of bioactive compounds from FBPs are sophisticated. Fruits are often contained different types of biological hazards i.e., dust, mud, pesticides, mycotoxins, anti-nutritional constituents, bacterial constituents, heavy metals, and biogenic amines that can cause severe health injury to humans (Shahbaz et al. 2022). For example, thiabendazole, carbaryl residues were found in mango peel extract (Teixeira and Poppi 2020), carbendazim, abamectin, cypermethrin residues were found in orange by-products (Li et al. 2012), the trace of heavy metals like lead, cadmium was noticed in orange, pomelo, mandarin, lemon and grapefruit’s peel (Czech et al. 2021). The presence of anti-nutritional compounds like oxalates, tannins, saponins, alkaloids, and cyanidin may cause an adverse effect in digestion, alter the absorbability of minerals, and inactivate the effect of vitamins (Munekata et al. 2022). Moreover, the by-products recovered from those fruits are not always available in ready-to-use conditions. It needs several pretreatments such as drying, disinfection, and size reduction before utilizing it in foods or taking itself as food. The drying, disinfection, and size reduction processes can minimize microbial contaminations, and improve the storability, the extractability (Michalska et al. 2017). These factors are taken into account during the isolation and utilization of natural bioactive complexes from FBPs.

This review offers an inclusive summary of bioactive substances discovered in FBPs and their potential uses, along with information on their obstacles and barriers, quality and safety evaluations, sensory characteristics, and solutions. From a technological viewpoint, the recent advancements in the sustainable use of natural bioactive substances from fruit wastes are also presented. This review is anticipated to be helpful to consumers who are concerned about their health as well as the dependent businesses as it is designed considering an eco-friendly environmental concern-oriented approach and taking into account circular economy notions.

## Bioactive compounds from fruit by-products (FBPs)

The bioactive compound can be defined as essential (e.g., carbohydrate, protein, lipids) and non-essential (i.e., polyphenols, carotenoids, flavonoids, anthocyanins, vitamins, and essential oils) compounds that occur naturally, are part of the food chain, and may be demonstrated to offer health advantages for humans. A significant quantity of non-essential bioactive substances can be found in plants. Most of those compounds exhibit various functions in plants. For instance, due to their bitterness and astringency they safeguard fruits and vegetables from early eating (Padayachee et al. 2017), attract insects and birds due to their attractive color formation (Sharma et al. 2021), and have biological activities (i.e., antioxidant, antimicrobial, anti-inflammatory and anticancer) in human health when consumed (Srinivasan et al. 2007; Boots et al. 2008; Hasan et al. 2019). Bioactive compounds are also signified as nutraceuticals which reflect their presence with biological significance in the human diet. However, FBPs are rich in high-value bioactive compounds and dietary fibers presented in Table 1 as an example; accordingly, the discussion in the following sections will center on the existence of these substances and how they bring value to foods.

**Table 1:** Bioactive components present in some different fruit by-products

Fruit	Fractions	Value-added compounds	Bioactivity	Application (Food/Non-Food)	References
Mango (*Mangifera indica*)	Peel	- Anthocyanins (mg/g DW): 0.03 to 0.39 - Carotenoids (mg β-carotene eq/g): 1.22 to 2.01 - Phenolics (mg GAE/g): 46.41 to 133.71	Antioxidant and anti-proliferative activity	Drug Carrier, Edible coating	(Mugwagwa and Chimphango 2019)
	Peel	- Total dietary fiber (%): 6.5 to 20.7 - Phenolics (mg GAE/g): 540 to 4500 - Carotenoids (mg/g DW): 17 to 247	Antioxidant Activity	Soft dough biscuit	(Ajila et al. 2008)
	Peel	Total phenolic content (mg GAE/100 gm): 220.33 to 757.8	Antioxidant Activity	Bread,	(Pathak et al. 2016)
	Peel	Carotenoids (µg/g DW): 4.64 to 84	Antioxidant Activity	Macaroni	(Ajila et al. 2010)
	Kernel	- Total phenolic content (mg GAE/100 gm DW): 72.1 - Total dietary fiber (g/100 g extract DW): 3.2	Antioxidant Activity	-	(Mutua et al. 2016)
Pomegranate (*Punica granatum*)	Peel	- Total Phenols (mg GAE/g DW): 1.17 to 10.59 - Total Flavonoids (mg QE/g dw): 0.54 to 7.19	Antioxidant activity	Cod Stick	(Panza et al. 2021)
Banana (*Musa acuminata*)	Peel	- Dietary fiber (%): 13.26 to 36.74 - Total Phenolics: 3.21 to 5.28 mg GAE - Insoluble dietary fiber (%): 8.43 to 26.87 - Soluble Dietary fiber (%):4.83 to 9.78	Antioxidant activity	Cookies	(Arun et al. 2015)
		Phenolics (mg GAE/kg yogurt): 244.85 to 522.65	Antioxidant and anti-diabetic activity	Yogurt	(Kabir et al. 2021)
Guava (*Psidium guajava*)	Seeds, skin, and pulp leftovers	- Phenolics (mg/g DW): 0.12 - β-carotene (μg/g DM): 7.91 to 56.07 - TDF (g/100 g extract DW): 89.8	Antioxidant activity	Fermented milk, cupcakes	(Casarotti et al. 2018)
Papaya (*Carica papaya*)	Peel	- Total Phenolics (mg GAE/g DW): 12.27 to 36.82 - Total flavonoids (mg QE/g DM): 0.60 to 0.91	Antioxidant and antidiabetic activity	–	(Islam et al. 2021)
	Seed	- Total Phenolics (mg GAE/g DW): 6.81 to 20.41 - Total flavonoid content (mg QE/g DM): 0.59 to 1.05	Antioxidant and antidiabetic activity	–	(Islam et al. 2021)
Pineapple (*Ananas comosus*)	Peel	- Phenolics (mg GAE/g DM): 15.69 to 26.84 - Flavonoid (mg QE/g DM): 1.56 to 2.68	Antioxidant and antidiabetic activity		(Islam et al. 2021)
Citrus fruits (*Citrus spp.)*	Seed	- Phenolics (mg GAE/g DW): 0.68 to 2.11 - Flavonoids (mg CE/g DW): 1.31 to 2.52 - Tannins (mg CE/g DW) 0.12 to 0.37	Cytotoxic activity and antioxidant activity	–	(Castillo-herrera et al. 2015)
Grape (Vitis vinifera)	Peel	- Total anthocyanin content (mg malvin chloride/g DW): 107 to 741.9 - Total phenol content (mg GAE/g DW): 177.1 to 254.6	–	Muffins	(Mildner-szkudlarz et al. (2016)

### Phenolic compounds

Phenolic compounds represent a varied group of biomolecules considered to be secondary metabolites found in plants. Chemically, molecular structures with an aromatic and benzene ring, as well as one or more hydroxide groups are called phenolic compounds; these compounds have derivatives with useful properties (glycosides, esters, methyl esters, etc.) (Barba et al. 2014). The wastes of fruit and vegetable are an abundant source of phenolic composites. In a study, Hernández-Carranza et al. (2016) demonstrated a substantial amount of total phenolic compounds in banana peel (304 to 50 mg of GAE/100 g DW), orange peel (391to 689 mg of GAE/100 g DW), and apple pomace (227 to 394 mg of GAE/100 g DW). The content of total phenolic may vary by different times, temperatures, solvents, and methods during extraction. Islam et al. (2021) assessed the by-products of different varieties of banana, jackfruit, mango, papaya, pineapple, papaya, and litchi in different extraction methods. The waste products (peel, seed, rags, and core) from those fruits demonstrated a significant amount of phenolic content, with values ranging from as low as 5.04 mg GAE/g DM to as high as 222 mg GAE/g DM. The pressurized hot-water extraction approach seemed to be more efficient than the organic solvent and enzyme-assisted extraction approach. Similar results were discovered in a study of bound and soluble phenolics in the peel and seed of six distinct tropical fruits: mango, logan, passion fruit, dragon fruit and rambutan (Nguyen et al. 2019). They found that rambutan peel had the highest concentration of soluble phenolic compounds (12.68 0.76 g/100 g DM) compared to mango seed (8.95 0.36 g/100 g DM). Moreover, it's noteworthy to notice that rambutan peel also exhibited the highest concentration of bound phenolic compounds (11.70 1.61 g/100 g DM) than other by-products. In that finding, the major phenolic compounds in soluble nature were geraniin, ellagic acid, and galloylshikimic acid, while the bound fraction was dominated by ellagic acid, with quercetin hexoside and gallic acid also present in significant amounts. However, among thousands of phenolic compounds, gallic acid, catechin, epicatechin, hydroxycinnamic acids, ferulic acid, quercetin, caffeic, procatechinic, syringic, vanillic acid, dopamine, hydroxy-tyrosol, tyrosol, cyanidin glycosides, L-dopa, p-coumaric acid, syringic acid, mangiferin, rutin, etc. are frequently identified in different FBPs. These mentioned phenolic compounds have strong antioxidant and antibacterial properties and may be used as food preservatives. For example, ferulic acid has potential antioxidant, antibacterial, anti-inflammatory, and anticancer properties, it functions as a skin protection agent, a preservative, and a cross-linking agent (Srinivasan et al. 2007). There have been claims that quercetin offers positive health effects against many illnesses and aging (Boots et al. 2008). A study demonstrated that pomegranate by-products improved the product quality of ready-to-cook cod sticks from a nutritional perspective as well as prolonged their shelf life by about three times (Panza et al. 2021). Another study claimed that the banana peel extract used in the manufacturing of bioactive compound-rich yogurts has been a success with promising antioxidant activity and extended shelf life (Kabir et al. 2021). Nevertheless, the total amount of phenolics in different FBPs and their application in developing healthy food products to have functional benefits are summarized in Table 1.

### Carotenoids

Carotenoids are the biological, fat-soluble compounds found in different FBPs along with, plants, algae, and photosynthetic bacteria. It generally works as a pigment of the biological body it belongs. Plants, vegetables, and fruits exhibit bright yellow, red, and orange colors with the help of it. Over 1,100 types of carotenoids are found in the different investigations (Yabuzaki 2017). All of them are divided into two categories: xanthophylls, which contain oxygen; and carotenes, which are completely hydrocarbons and contain no oxygen (Langi et al. 2018). In plants and algae, carotenoids provide two crucial functions: first, they absorb light for photosynthesis and second, provide photoprotection via non-photochemical quenching (Swapnil et al. 2021). But carotenoid works as a source of antioxidant when consumed by human or other animal species. It can protect from various diseases and enhance the immune system (Arihara 2014; Leoncini et al. 2015; Mitra et al. 2021).

Researchers have found carotenoids to be very common in FBPs. Mango, tomato, pineapple, jackfruit, guava, palm, banana, litchi, and so many other FBPs are analyzed and total or individual carotenoids were found. In a study, tomato pomace exhibited 1325 mg lycopene eq/g DW extract of carotenoids, whereas grape, olive, and pomegranate pomace displayed 7.0, 18.7, and 122.9 mg lycopene eq/g extract DW, respectively (Andres et al. 2017). Among passion, orange, and guava FBPs, the highest amount of carotenoids was found in passion FBPs (56.07 µg β-carotene and 28.57 µg lycopene/g extract DW) and the lowest amount of carotenoids were found in guava by-products (7.91 µg β-carotene and 4.7757 µg lycopene/g extract DW) by the study of Casarotti et al. (2018). Wang et al. (2008) observed the range of total carotenoid content was from 0.021 to 2.04 mg β-carotene eq/g DM extract among eight kinds of citrus fruits. The exocarp of fruits i.e., skin peel, pith, rind, etc. is known to be a better carrier of carotenoids than the other by-product of fruits i.e., seed, core, rag, axis, shell, etc. For example, Knoblich et al. (2005) reported higher carotenoid content in tomato peel (734.0 µg of lycopene/g DM) than in seed (130.0 µg lycopene/g DM). They also noticed carotenoids i.e., α-Carotene, Zeaxanthin, Lutein, and cis-β-Carotene were found greater in the peel than in the seeds. According to Šovljanski et al. (2022), the carotenoid content of horned melon pulp ranged from 0.81 to 1.14 mg β-carotene/100 g of extract, whereas peel ranged from 312.51 to 332.01 mg β-carotene/100 g of extract. The exocarp shows even higher carotenoid content than the pulp or flesh of the fruits.

Many food producers use carotenoids (i.e., lycopene, β-carotene) as colorants. But, it poses antioxidant qualities that can extend the shelf life of foods and act as a precursor of an essential vitamin, vitamin A (Strati and Oreopoulou 2014). There are many potential applications of this bioactive obtained from fruits; the food industry's impending usage of it as a food additive is still the most desired to meet customer satisfaction as natural food ingredients (Langi et al. 2018). Several studies were initiated to investigate the potential effect of natural carotenoids on human consumption. As a practical substitute of butter in the production of processed cheese, Bakry (2021) assessed the possible applications of lycopene oil extracted from tomato peel waste. They specifically compared cheeses prepared just with butter to cheeses made with lycopene oil added at various concentrations. The enhancement of lycopene content from lycopene oil in cheeses, antioxidant activity, meltability, and taste properties suggest that the replacement may be useful for decreasing saturated fats in dairy products. Ajila et al. (2010) and Ayala-Zavala et al. (2011) noticed that mango peel (4530 µg β-carotene /100 g DM) powder contains higher carotenoid than pulp (3092 µg/g). So, it could be an option to replace pulp with peel as a good source of carotenoid in preparing foods. Accordingly, Ajila et al. (2010) evaluated a feasibility study on macaroni incorporated with mango peel powder. This incorporation not only enhanced carotenoid content (4.64 to 84 µg/g DW) and storability but also improved organoleptic properties such as the color, taste, and texture of the macaroni.

### Dietary fibers

Dietary fibers are essentially carbohydrate polymers such as pectin, lignin, cellulose, and hemicellulose that provide structural rigidity of plant cell wall. They are classified into two classes based on their dissolvability in water: soluble dietary fiber (SDF) and insoluble dietary fiber (IDF). Soluble dietary fibers include mucilage,, gums, and pectin, whereas insoluble dietary fibers include lignin, cellulose, and hemicellulose (Hussain et al. 2020). Two major dietary fiber health claims have been acknowledged by the FDA. Their first statement is that the consumption of good levels of dietary fiber along with a low intake of fat diminishes the risk of cancer (U.S. Food and Drug Administration 2019). For instance, fibers from diverse sources including fruits, vegetables, and/or grains have been shown to minimize multiple types of cancer, including colorectal, oral, laryngeal, breast, prostate, and small intestinal (Williams et al. 2017). The second claim is that diets with high in dietary fiber and low in saturated fat and cholesterol are less likely to cause cardiovascular illnesses (U.S. Food and Drug Administration 2019). Obesity, hypertension, type 2 diabetes, hypercholesterolemia, and hyperlipidemia are the most prevalent risk factors for cardiovascular disease (Petrie et al. 2018). Numerous studies have suggested a link between increased dietary fiber intake and a reduced risk of diabetes, obesity, hypertension, gastrointestinal illnesses, stroke, and coronary heart disease (McRae 2017; O’Grady and Shanahan 2018).

Fruit by-products and waste can be a valuable source of dietary fiber. Many studies claimed a trace of dietary fiber in FBPs. For example, Casarotti et al. (2018) characterized guava, orange, and passion FBPs and investigated the impact of using these FBPs in probiotic fermented goat milk and fermented items made from cereals. According to their studies, the total dietary fibers in the by-products of guava, orange, and passion fruits were 89.80, 58.20%, and 64.20%, respectively. Tlais et al. (2020) also noticed 69%, 64.3%, and 67%, of dietary fiber in the peel of banana, mango, banana, and citrus fruits, respectively, and the majority of the proportions were insoluble dietary fiber. In another research, Karaman et al. (2017) isolated dietary fibers from the seeds of lemon, orange, and grapefruits, and the soluble dietary fibers ranged from 4.59 to 7.95% and the insoluble dietary fibers were 75.95 to 82.24%. It has been shown that the passion fruit peel contains a high quantity of total dietary fiber ranging between 57.93 and 71.71/100 g DM (Hernández-Santos et al. 2015). It has been discovered that adding this fiber from the passion fruit peel to yogurt enhances its fatty acid composition, microbiological stability, and conjugated linoleic acid content (Espírito-Santo et al. 2012). Numerous studies have demonstrated that dietary fiber in varying levels may improve food quality (Table 1). For instance, Saini et al. (2019) noticed that noodles formulated with banana flour rich in dietary fiber were highly nutritious and well-liked by consumers. Cakes made with wheat flour and 25% apple pomace mixture showed greater consumer approval ratings and had a more pleasing fruity flavor (Sudha et al. 2007). According to Ajila and Prasada Rao (2013) and Hussain et al. (2020), mango peel offers a significant level of dietary fiber that can be employed as a component of functional food items.

### Flavonoids

A class of polyphenolic chemicals called flavonoids are found everywhere in plants and comprise a broad class of natural goods. About 8000 distinct flavonoids have been discovered so far, most of which are found on the surfaces or in the cells of different plant organs (Jan and Abbas 2018). Flavonoids are thought to be significant nutritional components for humans and are found in many different edible plant species. It is one of the most prevalent and numerous categories of secondary metabolites, which is incredibly beneficial to humanity due to its many biological and physiologically active components.

Several flavonoids are readily identifiable as floral pigments in the majority of angiosperm species. However, they are present in various plant portions and not just in flowers; peel, rind, seed, and rag can contain ample flavonoids such as chalcones, flavanols, and isoflavones. Current research and development trends have attempted to find total and individual flavonoids as well as their applications on health advantages (Table 1). In such a study, Fatemeh et al. (2012) reported that a specific variety of banana peel contains 39.01 to 389.33 mg CEQ/100 g DM. Ajila and Prasada Rao (2013) identified three types of bound flavonoids in four different varieties of mango. Among them, kaempferol (ranged from 10.29 μg/g to 52.73 μg/g extract) and quercetin (ranged from 5.84 μg/g to 56.83 μg/g extract) comprised the major part of it. Similar outcomes were observed by Wang et al. (2008) who claimed that orange peel contained rutin, quercetin, and kaempáerol. Hernández-Carranza et al. (2016) found the highest amount of total flavonoid content of 752, 667, and 586 mg of catechin/100 g DW in banana peel, orange peel, and apple pomace, respectively. A significant amount of the total flavonoid content was noticed in litchi (peel: 48.67 mg QE/g DM seed: 30.57 mg QE/g DM), mango (peel: 8.61 mg QE/g DM seed: 6.28 mg QE/g DM), and banana (peel: 16.44 mg QE/g DM seed: 10.79 mg QE/g DM) (Islam et al. 2021).

## Necessity of physical pretreatment before utilization of FBPs

This is a vital step in the process of turning the fruit "waste" into a viable by-product ingredient. In this stage, producing an ingredient that is microbially stable and minimizes bioactive losses (such as flavonoids, carotenoids, and phenolics) and aids in the development of an ingredient that has a wide range of health-improving advantages (Kabir et al. 2021; O’Shea et al. 2012). The development of these components from by-products involved numerous techniques. Several FBPs can be enhanced in terms of storage and handling by drying, reducing particle size, and densifying (Taghian Dinani and van der Goot 2022). The wet/moist by-products frequently begin to deteriorate chemically and biologically while being stored (Paulino et al. 2020). Drying is required if the item cannot be kept or processed further while still wet. If the by-product is particularly bulky or contains larger particles, size reduction by trimming or crushing may be required. Densification via baling, briquetting, or pelletizing may be necessary to minimize transit and storage costs if the substances to be treated is delivered to a facility for conversion or is kept on hand for year-round supply. These pretreatments will be spread in the following portions.

### Necessity of drying, size reduction, densification, and concentration

The high moisture content in FBPs makes them vulnerable to microbial contamination and chemical degradation during storage or if not handled promptly (O’Shea et al. 2012). The wet-FBPs are produced in great volumes during specific seasons, making it impossible to treat a large quantity of them right once. As a result, their direct industrial application is not suitable (Taghian Dinani and van der Goot 2022). A wide range of pretreatment techniques and parameters are used during the processing of by-products, but drying is one of the most common techniques. By choosing the right pretreatment parameters, drying can minimize microbiological contamination, chemical deterioration, and losses of bioactive ingredients (Michalska et al. 2017). The reduction of chemical changes owing to water removal is one of the major benefits of drying that makes items more stable. Microbial contamination can be drastically decreased with 24 h of drying at 60℃, according to research by Reissner et al. (2019). Due to its application in later phases of food processing, drying can also assist in resolving various environmental (contamination), health, and safety issues (Özcan et al. 2021). Özcan et al. (2021) noticed that the application of drying techniques had an impact on the amount of phenolic chemicals, fatty acids, carotenoids, and flavonoids in orange by-product compositions. Higher temperatures and lengthy drying times typically result in low-quality dried products, hence numerous innovative research studies have reported the use of innovative drying techniques (Badaoui et al. 2019). Nevertheless, the choice of a suitable drying technique is the last step in creating a balance between the economics of value-added ingredients extraction, dried by-products quality, and food processing (Majerska et al. 2019).

FBPs frequently come in sizes that are unsuitable for use and do not permit effective extraction on an industrial scale, thus they must be reduced in size (Gerschenson et al. 2015). The size reduction process, an important unit operation in the food business, involves breaking down materials into smaller pieces. Particles of a certain size and shape are created by applying various forces. The first is grinding, a common physical pre-treatment process that reduces particle size and raises the contact surface area (Fuso et al. 2021). A smaller size means a larger surface area, which enhances water absorption, increases solubility, releases flavor, creates a softer mouthfeel, and increases the FBPs and solvents contact surface area throughout the extraction process. Due to the huge specific surface area of small particles, the reaction rate of the product is considerably boosted in the biological and chemical processes (Li et al. 2012). In general, the reduction of size can be carried out in a dry or a wet condition, although wet crushing uses extra energy when compared with dry grinding (Arshadi et al. 2016). However, the optimized size reduction method can increase the yields of particularly costly value-added components extraction, so before the main extraction process, it is advised to perform this size reduction.

The densification method is useful for overcoming certain biomass application limitations include uneven particle size and shape, poor density, and high transportation costs (Sarangi et al. 2018). FBPs are typically clumpy and have a low density. Seasonal fluctuations affect the availability of these residues, posing a problem for storage and handling (Vaish et al. 2022). Due to their low density, these by-products are difficult to convert into value-added products, especially when they need to be transported or held for an extended period for storage or in a transformation facility. Therefore, food by-product densification is necessary to enable enhancing the extraction of precious substances (Taghian Dinani and van der Goot 2022). The processes of densification can be categorized as extrusion, baling, palletization, and briquetting, which are executed with a screw press, roller press or piston press, pelletizer, and bailer (Tumuluru et al. 2011). Extrusion, pelletizing, briquetting, pelletizing, and baling are typical techniques for densifying various types of biomass. Densification has a number of benefits, such as increased efficiency in handling and transport, more uniform and dense feedstock through controlled particle size distribution, higher quality composition through fractionated structural components, and conformity to conversion technology and supply system specifications (Arshadi et al. 2016).

The food industry faces significant financial losses due to oxidative reactions in fatty foods during storage. Synthetic antioxidants like BHA, BHT, and TBHQ are added to slow down deterioration, but concerns about their harmful effects have led to customers seeking natural plant-based substitutes (Agregán et al. 2017). Natural extracts, such as polyphenols and carotenoids, have been successfully used in complex foods like dairy, bakery, beverage, and meat products (Kabir et al. 2021; Marcillo-Parra et al. 2021; Trigo et al. 2020). Excellent bioactive substances with potential antioxidant action, like polyphenols and carotenoids, can be found in FBPs (Islam et al. 2021). After extracting the valuable ingredients from by-products, it has to be purified and concentrated to increase their effectiveness. The final polyphenol extract requires drying since it completes the extraction process by eliminating the rest of solvent and concentrating the extract. (Rajha et al. 2014). During vaporization, the solution is heated to eliminate the solvent; in this case, rotary evaporators are commonly used to create concentrated solutions, preserving enzyme activity, recovering solvents, and stabilizing the product (Kabir et al. 2021). Polyphenols may lose their biological activity if the product is exposed to moderately high heat for an extended period (Rajha et al. 2014). In this context, membrane filtration techniques offer a cost-effective, easy-to-maintain, and selective alternative to conventional technologies for preserving the biological activity of polyphenols. They do not require chemical or mass extraction agents, and ultrafiltration is commonly used for separating useful components from solutions. To isolate and concentrate useful components from a solution, ultrafiltration is frequently utilized (Rodrigues et al. 2022).

### Recovery of phytochemicals using the traditional approach

The recovery of phytochemicals using traditional methods involves extracting bioactive compounds from plant materials using various techniques that have been practiced for centuries. These methods are often simple and require minimal equipment. Some common traditional methods for phytochemical recovery include maceration, decoction, infusion, distillation, cold pressing, and soxhlet extraction (Bitwell et al. 2023). Maceration is the process of soaking plant material in a solvent such as water, alcohol, or oil in order to dissolve the bioactive chemicals into the solvent (Kowalczewski and Zembrzuska 2023). During this soaking period, the solvent gradually breaks down the plant cell walls and extracts the phytochemicals from the plant material. The mixture is then periodically shaken to facilitate extraction. The choice of solvent, duration of maceration, and the temperature at which the process occurs can all influence the quality and quantity of phytochemicals extracted (Vincent et al. 2022). After the maceration process is complete, the liquid extract can be filtered to remove the solid plant material, and the resulting solution can be used for various purposes.

In the method of decoction, plant materials are boiled in water for an extended period. The heat helps release the phytochemicals into the water, and the resulting liquid is then strained and used. It's worth noting that the effectiveness of decoction can vary based on factors like the plant material used, the boiling time, and the water-to-plant ratio (Manousi et al. 2019). Similar to making tea, infusion involves pouring boiling water over plant materials and letting them steep for a certain period. The recovery of phytochemicals through infusion is a way to harness the potential health benefits of these compounds (Alara et al. 2021). Different plants contain various types and concentrations of phytochemicals, and these compounds have been linked to a wide spectrum of positive effects on human health, including anticancer properties, anti-inflammatory, and antioxidant activities, among others (Rodríguez-Yoldi 2021). Another traditional extraction method is distillation, which is used to extract volatile chemicals from plant materials such as essential oils. Steam distillation is a common method where steam is passed through the plant material, carrying the volatile compounds with it. The steam is then condensed to separate the essential oil (Maharaj and McGaw 2020). There are various distillation procedures that can be used to recover phytochemicals, including vacuum distillation, steam distillation, short-path distillation, and simple distillation. The choice of distillation method depends on the specific phytochemicals that are trying to extract, their stability under heat, and the desired purity of the final product (Kumar et al. 2023). It is essential to emphasize that while distillation can be effective for recovering phytochemicals, it may not be suitable for all types of compounds. Some compounds might degrade under the high temperatures used in distillation, and alternative extraction methods, such as solvent extraction or cold pressing, might be more appropriate in those cases.

Cold pressing is often preferred because it maintains the natural flavors, aromas, and nutritional compounds of the source material. In the context of phytochemicals, which are bioactive compounds naturally occurring in plants and have potential health benefits, cold pressing is considered a gentle extraction method. Many phytochemicals are heat-sensitive and can degrade when exposed to high temperatures, so using cold pressing helps preserve these compounds (Al Ubeed et al. 2022). Soxhlet extraction is a common technique used to extract and recover various compounds, including phytochemicals, from solid materials, especially plant materials. This technique is particularly useful when there is a need to extract compounds that are not easily soluble in the extraction solvent at room temperature, or when you want to perform exhaustive extractions to maximize the yield (Bitwell et al. 2023). Soxhlet extraction is utilized extensively in various fields, including natural product chemistry, pharmaceuticals, and food science, to isolate and purify bioactive compounds from plant materials. It is important to note that while Soxhlet extraction can yield high-quality extracts, it can also be time-consuming and require a relatively large amount of solvent, which might not be environmentally friendly (Rahmana et al. 2023).

**Fig. 2:**
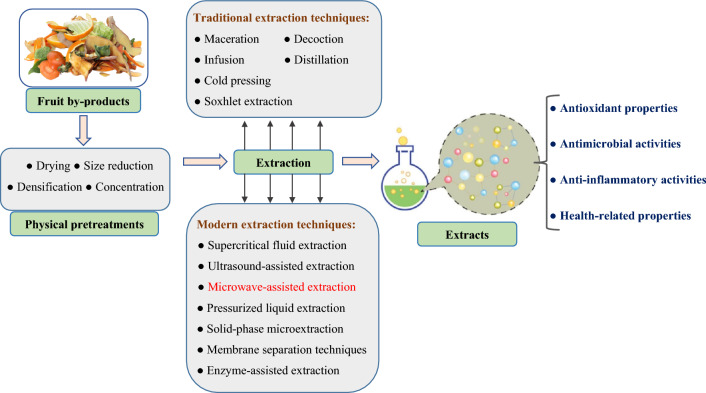
Pretreatments and recovery of phytochemicals from fruit by-products using both traditional and modern approach

Traditional extraction methods might have limitations in terms of efficiency, selectivity, and reproducibility but are still used in various cultures. Alternative modern extraction techniques, such as ultrasound-assisted extraction, supercritical fluid extraction, and chromatography have been developed to address some of these limitations. These methods often provide higher yields, shorter extraction times, and better control over the extraction process. However, traditional methods continue to be relevant for small-scale applications, home remedies, and cultural practices (Fig. 2).

### Recovery of phytochemicals using the modern approach

The modern approach involves the utilization of advanced techniques and equipment to extract and purify bioactive composites from plant sources. The recovery process aims to isolate these valuable phytochemicals for various applications, such as pharmaceuticals, nutraceuticals, cosmetics, and food additives. The modern approaches include supercritical fluid extraction (SFE), ultrasound-assisted extraction (UAE), microwave-assisted extraction (MAE), pressurized liquid extraction (PLE), solid-phase microextraction (SPME), membrane separation techniques (MST), and enzyme-assisted extraction (EAE) (Nguyen et al. 2023; Domínguez et al. 2021).

SFE is a sustainable alternative to traditional extraction systems for removing natural chemicals like essential oils, carotenoids, flavonoids, and fatty acids from plant constituents (Uwineza and Waśkiewicz 2020). Carbon dioxide (CO_2_) is an outstanding solvent for SFE due to its chemical inactivity, economic accessibility, separability, non-toxicity, and food-grade status. Nonpolar gas-like and liquid-like supercritical CO_2_ can extract heat-sensitive compounds (Gan and Baroutian 2022). To get the best outcomes during SFE, proper temperature, time, and pressure control is crucial to ensure optimal results (Ahangari et al. 2021; Rochfort et al. 2020). The primary drawbacks are still its expensive price and the challenge of carrying out continuous extractions (Fomo et al. 2020). A mechanical method known as UAE uses sound waves, frequencies, and amplitudes to encourage the breakdown the cell barriers and the discharge of cell substances. Due to its impact on the solvent/sample ratio, temperature, and transport mass, this method shortens the extraction time, works with thermolabile and unstable chemicals, enhances the efficiency of extractions, and uses less energy and solvent (Mehta et al. 2022). However, ultrasound energy can occasionally cause unwanted changes in bioactive compounds due to the formation of free radicals.

MAE is a recent method for obtaining natural substances using microwaves and solvents that involves heating the solvent and plant tissue to enhance extraction kinetics (Bitwell et al. 2023). The microwave-to-heat energy conversion is proportional to the dielectric constant of the solvents. The technique preserves the biological activities of extracts, improving antioxidant activity and enhancing color quality, and phenolic components (Alam et al. 2023). MAE has been used to obtain various phytochemicals, including saponins, polyphenolic antioxidants, sterols, and flavonoids. Microwave power, extraction time, and temperature significantly influence extraction efficiency in MAE (Wong and Nillian 2023). MAE is user-friendly and versatile, allowing for shortened extraction times and reducing complexity in food extraction. Recent applications of MAE in extracting phytochemicals highlight its versatility and potential in food extraction. Moreover, the recoveries of phytochemicals through EAE method have limitation due to the requirements of aqueous phase during the extraction process (Kumar et al. 2021).

PLE is a sophisticated extraction method that uses solvent extraction at high temperatures and pressures, maintaining the solvent’s liquid condition throughout the process. Higher extraction yields and less solvent consumption for sample preparation are obtained using this method, which is also known as high-pressure solvent extraction, pressurized fluid extraction, accelerated solvent extraction, pressurized hot solvent extraction, subcritical solvent extraction, and high-pressure high-temperature solvent extraction (Barp et al. 2023). PLE is versatile and can be used with a wide range of solvents, with water being the most environmentally friendly option. The most important parameters in PLE method development include extraction temperature, extraction pressure, extraction time, and the type of extraction (dynamic mode, static mode, or a combination of both) (Perez-Vazquez et al. 2023). Every type of sample requires optimization of the various elements influencing the extraction process, and a full factorial experimental design might be helpful in determining the best extraction conditions (Morshed et al. 2020). PLE is popular in the food and pharmaceutical industries due to its speed, less toxicity, and environmental benefits. The primary constraints of PLE are the substantial financial investment required for the necessary equipment and the meticulous control of extraction parameters, including pressure and temperature. Some investigations have shown the utilization of mild temperatures during PLE may result in the deterioration of thermolabile chemicals that may be intensified with extended treatment times (Picot-Allain et al. 2021).

Solid-phase microextraction (SPME) is easy to use extraction technique that involves dispersing the solid phase in a small amount of extracting phase and exposure to the sample for a precise period (Jalili et al. 2020). It has benefits such as simplicity, and automation, and is effective for analyzing bioactive compounds in low concentrations in foods. SPME can also reduce solvent clearance issues and facilitate research on small samples, such as single cells (Usman et al. 2022). To isolate and purify phytochemicals from waste streams, nanofiltration, microfiltration, and ultrafiltration separation methods are utilized, which requires no organic solvents. These processes are suitable for getting phenolics out of waste streams from the food industry using different membrane types (Papaioannou et al. 2022; Tapia-Quirós et al. 2022). The EAE method uses enzymes commonly lactase, protease/lipase, lipase, phospholipase, pectinases, cellulases, and hemicellulases to release phytochemicals found in cells of aromatic and medicinal plants (Ebrahim 2023; Fărcaș et al. 2022). These enzymes can destroy or disrupt the cell walls and membranes of plants, resulting in a more effective release and extraction of active chemicals. This process decreases the use of solvents and energy, making it a greener and more efficient alternative to conventional extraction methods (Islam et al. 2021; Islam et al. 2023a, b). However, it has drawbacks such as high costs, difficulties in industrial-scale applications, and enzyme limitations in hydrolyzing plant cell walls.

These modern approaches offer various benefits, including increased efficiency, reduced solvent usage, higher selectivity, and improved yield of phytochemicals. However, the method selection criteria depend on the specific phytochemicals of interest, the plant source, and the intended application of the recovered compounds. Therefore, these modern approaches are still having some limitations include increased operational complexity, challenges in achieving industrial scalability, and higher costs. By combining strategies including process optimization, technology integration, scale-up strategies, economic consideration, and collaborative industry efforts, businesses can work towards overcoming the limitations associated with modern extraction approaches, ensuring a more efficient, scalable, and cost-effective operation.

### Recovery of value added products through biotransformation

In recent years, the field of biotransformation has gained significant momentum, owing to advancements in gene engineering and microbiology. Biotransformation is a cutting-edge technology that holds great promise for efficiently utilizing waste generated from bioresources to synthesize valuable products (Mishra et al. 2023; Vickram et al. 2023). One particularly promising avenue within biotransformation is the utilization of FBPs into high-value products, contributing to sustainability and resource optimization. Through biotransformation processes, enzymes or microorganisms can be employed to convert FBPs into bioactive compounds such as antioxidants, dietary fibers, enzymes, biofuels, and essential oils (Sadh et al. 2018). These transformed products can find applications in the food, pharmaceutical, and cosmetic industries. For example, mango fruit by-products can be used as a substrate for the production of pectinase from *Fusarium spp.* microorganisms (Kowalska et al. 2017). Biotransformation of fruit residues including mango (*Mangifera indica*), pineapple (*Ananas comosus*), grape (*Vitis vinifera*) fruit, orange (*Citrus sinensis*), and passion fruit (*Passiflora edulis*) via solid state bioprocess using *Lichtheimia ramose* to evaluate the mycelial growth and the enzymatic activities and their influence in the generation of molecules with potential use (de Andrade Silva et al. 2020). Therefore, the utilization of FBPs through biotransformation represents a promising avenue for sustainable and value-added product synthesis. This innovative approach not only addresses environmental concerns associated with fruit processing waste but also opens up new possibilities for economic growth and technological advancements in the field of biotechnology.

The integration of synthetic biology into the production of bioactive components from fruits has the potential to significant influence the status of FBPs utilization. By employing engineered microbial strains, researchers can design efficient and cost-effective pathways for the synthesis of specific bioactive compounds that are naturally present in FBPs (Guo et al. 2023; Kowalczyk et al. 2022). Research showed that several enzymes could be produced from fruit by-products by the application of microbial strains. According to the representation of Mahato et al. (2021), enzymes such as pectinase, cellulase, xylanase, amylase, and lipase produced from orange peels and pulps by the application of strains of *Aspergillus niger A-20* and *Chaetomium spp. (KC-06).* In another study presented that microbial strains including *Saccharomyces cerevisiae SDB, Aspergillus niger ANSS-B5, and Corynebacterium glutamicum CECT 690* used for the production of ethanol, citric acid, and glutamic acid, respectively, from date palm fruit by-products (Chandrasekaran and Bahkali 2013)*.* It is important to note that there are potential challenges and considerations, such as regulatory approval, public acceptance, and ethical concerns associated with the use of genetically modified organisms (Myskja and Myhr 2020). However, the integration of synthetic biology into the production of bioactive substances from FBPs has the potential to revolutionize the field, offering a more direct and cost-effective approach. Therefore, researchers and agro-processors should carefully analyze the implications of these technologies on current practices, considering both the benefits and challenges associated with this emerging paradigm shift in bioactive compound production.

## Safety assessment of valuable ingredients from FBPs

**Fig. 3:**
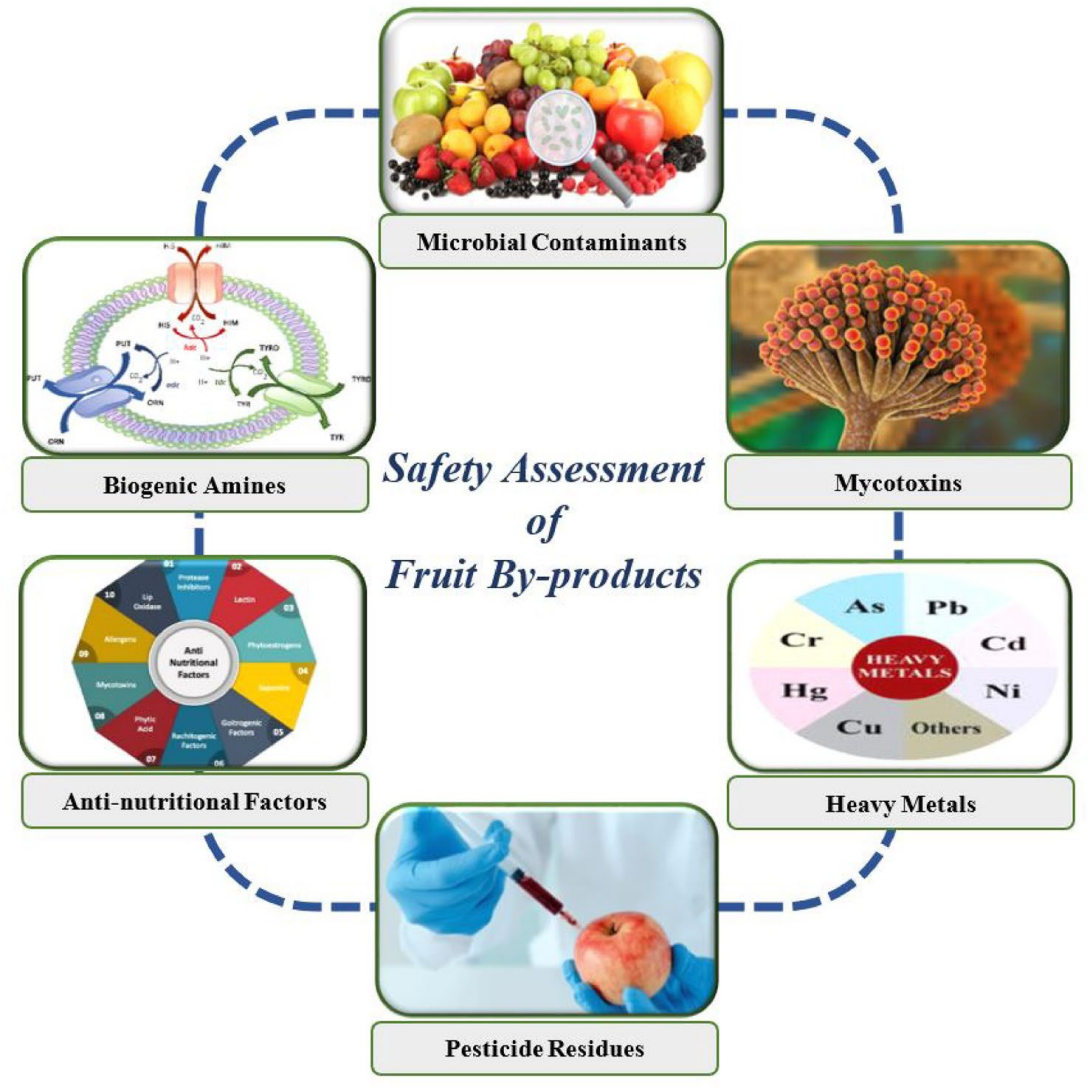
An overview of classification of safety indicators in fruit by-products

Food safety is a significant issue for trade in food products around the world. Therefore, the extraction and application of natural compounds from FBPs raise concerns about their safety (Taghian Dinani and van der Goot 2022). As already mentioned, FBPs are the richest source of bioactive substances, which have important antioxidant, anti-inflammatory, anti-proliferative, anti-diabetic, and antibacterial properties (Islam et al. 2021; Srinivasan et al. 2007; Boots et al. 2008). The use of those compounds derived from by-products faces various obstacles that can compromise product safety including potential pathogen contaminations, biological instability, greater water activity, high amounts of active enzymes, and the possibility of rapid auto-oxidation. Exclusively, the safety of bioactive compounds in FBPs can be cooperated through contamination with pesticides, mycotoxins, metals, toxic plants and so on (Fig. 3).

### Contaminants assessment

Reintroducing FBPs into the human food chain raises concerns about the possible persistence of pollutants in the valuable ingredients derived from FBPs. The most well-known contaminants referred to are pesticides, mycotoxins, anti-nutritional constituents, bacterial constituents, heavy metals, and biogenic amines (Vilas-Boas et al. 2021), some examples are presented in Table 2. Severe diseases are caused by all of these biological hazards and therefore, several considerations must be made before approving the FBPs as suitable valuable components.

**Table 2:** Example of potential contaminants found in FBPs

Contaminants	Type	By-products	References
Pesticides	Imidacloprid, Carbendazim, Abamectin, Cypermethrin, Prochloraz	Orange by-products	(Li et al. 2012)
	Thiram, Chlorpyrifos, Methyl parathion	Apple, grape, pear, mango, and peach fruit peel	(Liu et al. 2012)
	Thiabendazole, Carbaryl	Mango peel extract	(Teixeira and Poppi 2020)
	Cyprodinil, Diethomorph, Famoxadone, Dimetoathe, Diazinon	Grape skin extract	(Moncalvo et al. 2016)
Mycotoxin	Alternariol, Alternariol monomethyl ether, Tentoxin, Aflatoxins, Ochratoxin A	Berry FBPs	(Juan et al. 2017)
	Fumonisin B1	Orange peel	(Rasheed et al. 2020)
	Ochratoxin A	Grape skin extract	(Moncalvo et al. 2016)
	Tryptoquialanines A, Tryptoquialanines C	Orange peel	(de Vilhena Araújo et al. 2019)
Ant-nutritional contaminants	Saponins, Alkaloids, Tannins, Phytates, Oxalates	Rambutan FBPs	(Mahmood et al. 2018)
	Saponins, Oxalates, Phytates, Tannins	Melon by-products	(Silva et al. 2020)
Microbial contaminants	Proteobacteria, Actinobacteria, Bacilli	Banana, guava, mango, papaya, passion fruit peel	(Cruz et al. 2019)
Heavy metals	Lead, Cadmium	Orange, pomelo, mandarin, lemon, grapefruit peel	(Czech et al. 2021)
	Cadmium, Lead	Grape skin extract	(Moncalvo et al. 2016)
	Cadmium, Chromium, Copper, Lead, Zinc, Nickel, Manganese	Orange, yellow lemon, banana peel	(Saleem and Saeed 2020)
	Copper, Zinc, Cadmium, Aluminum	Sweet orange fruit peel	(Dhiman et al. 2011)
Biogenic amines	Ethanolamine, Ethylamine, Putrescine, Cadaverine	Grape skin powder and extract	(Moncalvo et al. 2016)

As a preventative measure against weeds, insects, and diseases, pesticides are frequently used in the field and after harvest for many types of fruits (Li et al. 2012). The valuable ingredients and extracts from FBPs that have been treated with pesticides during and after production will no longer be considered natural ones. The selection of an appropriate solvent for the extraction of valuable compounds is important because pesticides can selectively solubilize and even concentrate in the products (Vilas-Boas et al. 2021). Although the correct use of pesticides by maintaining concentration and limit does not cause harm to public health and the environment.

Mycotoxins are secondary metabolites that can be found in a wide variety of foods and feedstuff. They are produced by certain fungi and may impose food safety risks to human health due to their widespread distribution (Kumar et al. 2022). Aflatoxins, ochratoxin A, deoxynivaleonol, zearalenone, and fumonisins have received a lot of attention because of their prevalence and the wide range of health problems in both humans and animals. The human body can absorb mycotoxins through the skin, the lungs, or the digestive tract. Mycotoxins are a group of compounds that can be ingested by animals and humans and result in illness, decreased performance, or even death (Afsah-Hejri et al. 2013). Adverse health effects such as carcinogenic, teratogenic, immunosuppressive, or estrogenic effects can occur immediately after consuming mycotoxin-contaminated food or feed, or they can develop over time (Lai et al. 2017). The best prevention and control strategies for mycotoxin are not anticipated to be achieved by a single treatment but rather through a combination of good agricultural and manufacturing practice, suitable storage conditions, suitable environmental control, bio-safe post-harvest detoxifying methods, and proper quality assurance programs (Peivasteh-Roudsari et al. 2021; Vilas-Boas et al. 2021).

A large number of microbes may present in FBPs (Table 2) that result in a breakdown of protein leading to the formation of strong odors. Many different kinds of FBPs still contain enzymes that hasten or amplify spoiling events (Jayathilakan et al. 2012). The ability to grow or survive of microorganisms depends on the presence of the amount of water. The microbial contamination of FBPs is often attributed to foodborne pathogens, e.g., E. coli and Salmonella spp. (Taghian Dinani and van der Goot 2022) that comes from contaminated fertilizer which is mostly animal-based fertilizer (Vilas-Boas et al. 2021). Therefore, safety is a crucial criterion for the development of healthy food introducing FBP exhibiting no microbial contaminations. To satisfy those prerequisites, some of the strategies used to eliminate microbial contamination such as heat treatment or ionizing radiation; extracts obtained from FBPs that have been pasteurized or subjected to sterilizing filtration can affect the final product's quality (Cádiz-Gurrea et al. 2020).

Anti-nutritional factors are compounds that can present in FBP (Table 2) that block several pathways of dietary nutrient absorption (Taghian Dinani and van der Goot 2022). These substances have a negative influence on protein digestion and utilization (saponins, lectins, trypsin and chymotrypsin inhibitors); an unfavorable impact on carbohydrate digestion (amylase inhibitors, polyphenolic compounds, flatulence causes); and deactivate vitamins (Munekata et al. 2022). Phytate is a frequent anti-nutrient in the form of an insoluble compound with minerals such as calcium, zinc, iron, and copper resulting in a deficiency of those nutrients. As a result, several measures for managing FBP quality (such as the drying process) should be considered, heat treatment is the most typical treatment used for the reduction of these anti-nutritional continents to a reasonably safe level (Lai et al. 2017). However, heat treatment may not be effective for all anti-nutritional constituents; gamma-irradiation and ultraviolet can also be used for the reduction of anti-nutritional factors in samples. However, in some special cases, gamma and UV radiation may slightly reduce the vitamin C concentration, tocopherol, carotenoids, phenolic compounds, and anthocyanins (Taghian Dinani and van der Goot 2022).

The metal contaminants that are commonly detected in fruit waste (Table 2) especially are cadmium, chromium, copper, lead, mercury, nickel, and zinc (Dhiman et al. 2011; Czech et al. 2021); which are particularly disturbing due to their inherent toxicological concern when present in food supplements. This form of contamination may occur because of a single factor or as a combination of sources depending on the type of supplement (Costa et al. 2019). Factors that depend on contamination with metals in plant-based supplements are influenced by plant features, chemical soil composition, plant features, their growing conditions, fertilizers derived from animal sources, as well as a lack of purity, formulation/manufacturing, extraction methods, shipping, and storage conditions (Vilas-Boas et al. 2021).

Biogenic amines (Table 2) are nitrogen compounds with low molecular weight organic bases that are commonly present in a variety of foods and are generated and destroyed by particular bacteria through the enzymatic metabolism of certain free amino acids. Some precursor amino compounds can be decarboxylated, reductively amined, transaminated, or broken down by this enzymatic reaction (Vasconcelos et al. 2021). In addition to their potential toxicity, biogenic amines in food are of interest because they are used as a quality indication of freshness or spoilage and as a means of assessing the impact of microorganisms during food preparation (Verma et al. 2020). Biogenic amines, especially cadaverine, putrescine, and histamine are suggested as signs of spoilage of some foods, such as fruits, vegetables, meat, and fish. The amount and kind of biogenic amines produced are greatly regulated by the food's chemical composition, microbial flora, and other conditions that permit bacterial development during food processing and storage (e.g., food treatment before storage, moisture, temperature, food additives, ripening, and packaging). Low quantities of biogenic amines in food are not regarded as a serious health hazard (Saaid et al. 2009). However, When ingested in excess, they can have different physiological, pharmacological, and toxic effects on people, including vomiting, rashes, headaches, diarrhea, edema, hypertension, palpitations, and cardiac problems (Vilas-Boas et al. 2021).

Finally, it is expected that a HACCP system will be set up for the industrial use of value-added ingredients from FBPs. This will keep safe both the FBPs and the extracted ingredients from getting contaminated (Taghian Dinani and van der Goot 2022). All of these methods have the potential to boost the utilization of FBPs, which could increase producers’ financial gains and environmental safeguards at the same time.

### Anti-microbial potentiality assessment

One of the most important dangers to current global public health is antibiotic resistance, hence it is urgent to expand novel antimicrobial techniques to combat multidrug-resistant microbial strains (Saratale et al. 2020). Various antibiotics have been identified over the last decade and these antibiotics provide relief to humans suffering from a variety of life-threatening infections (Manganyi and Ateba 2020). Typically, disease prevention is accomplished through the use of chemicals that have negative consequences such as human health dangers and the development of microbial resistance to chemical use (Koch et al. 2021). Microbes have the genetic potential to develop resistance to medications used to treat illnesses. Currently, multidrug-resistant strains of disease-causing microorganisms are responsible for the worldwide increase in untreatable bacterial illnesses and mortality rates (Saleem and Saeed 2020). Because of such concerns, it is crucial to identify potentially viable safer, and natural alternatives is of importance. Utilization of antimicrobial compounds generated from plants with distinct chemical structures and novel modes of action as a barrier against multidrug-resistant strains of bacteria (Shin et al. 2018; Hasan et al. 2022). There are numerous options for producing antimicrobial medications from a variety of plants and their varied chemically diverse constituents (Saleem and Saeed 2020). Antimicrobial phytochemicals derived from FBPs can meet this demand, some examples are shown in Table 3. Recently, special emphasis has been placed on the usage of fruit processing by-products as a rich source of phenolic compounds with potential health and industrial applications (Yılmaz et al. 2019). Using FBPs therapeutically is an innovative concept, which is gaining acceptance because of their novel, natural, eco-friendly, and biodegradable functionality. These prospective economic sources for the extraction of antimicrobials can be employed to prevent diseases caused by pathogenic microorganisms.

**Table 3:** Antimicrobial activity of FBPs

FBPs	Target microorganism	Effects	References
Grape pomace extract	Escherichia coli, Staphylococcus aureus	- Exhibited anti-bacterial activity against both bacteria - Escherichia coli showed higher antibacterial activity when compared to the gram-positive bacteria Staphylococcus aureus	(Saratale et al. 2020)
Pineapple rind, Pomegranate peel, Orange peel, Avocado rind	Salmonella enterica serovar Typhimurium SL 1344	- The pomegranate peel has strong bioactive repelling capability against pathogenic microorganisms	(Mahadwar et al. 2015)
Orange, yellow lemon, and banana fruit peel	Gram-positive bacteria: Pseudomonas aeruginosa, klebsiella pneumonia, Serratia marcescens, Salmonella typhi, Proteus vulgaris, Escherichia coli Gram-negative bacteria: Aeromonas hydrophila, Listeria monocytogenes, Streptococcus pyogenes, Staphylococcus aureus, Enterococcus faecalis, and Lactobacillus casei Fungi: Penicillium citrinum, Aspergillus niger Yeast: Saccharomyces cerevisiae, Candida albicans	- Bacteria are more sensitive than all microbes - Gram-negative exhibited higher sensitivity - Bacteria were more sensitive against yellow lemon peel and had higher inhibition zone	(Saleem and Saeed 2020)
Pomegranate peel	Staphylococcus aureus, Escherichia coli, Klebsiella pneumonia, Bacillus coagulans, Bacillus cereus, Bacillus subtilis, and Pseudomonas aeruginosa	- Pomegranate peel exhibited a broad spectrum of antimicrobial effects having apparent inhibitory effects against the mentioned microorganisms	(Chen et al. 2020)
Pomegranate by-product	Staphylococcus epidermidis, Micrococcus kristinae	- Antimicrobial action against Staphylococcus epidermidis was demonstrated by phloretin and coutaric acid - The antibacterial action of punigratane is the strongest against Micrococcus kristinae	(Nazeam et al. 2020)
Pomegranate, apple, dog rose, cornelian cherry, hazelnut, white grapes, and red grapes FBP	Salmonella Enteritidis O:103, Pseudomonas fluorescens, Pseudomonas aeruginosa, Escherichia coli ATCC 35150, Escherichia coli DH5α, Serratia marcescens, Staphylococcus aureus ATCC 10527, Listeria monocytogenes NCTC 10527, Bacillus cereus DSM 350, Lactococcus lactis DSM 4366, Lactobacillus sakei DSMZ, Staphylococcus xylosus	- The antimicrobial activity against twelve foodborne pathogens and spoilage microorganisms was evaluated - Pomegranate and apple peels showed the highest inhibition of Staphylococcus aureus and Pseudomonas fluorescens	(Agourram et al. 2013)
Raspberry, blackcurrant, apple, and rowanberry by-products	Leuconostoc mesenteroides LUHS225, Lactobacillus plantarum LUHS122, Enteroccocus pseudoavium LUHS242, Lactobacillus casei LUHS210, Lactobacillus curvatus LUHS51, Lactobacillus farraginis LUHS206, Pediococcus pentosaceus LUHS183, Pediococcus acidilactici LUHS29, Lactobacillus paracasei LUHS244, Lactobacillus plantarum LUHS135, Lactobacillus coryniformis LUHS71, Lactobacillus brevis LUHS173 and Lactobacillus uvarum LUHS245)	- Broadest spectrum of pathogenic bacteria inhibition was shown by blackcurrant and apple by-products - The strongest inhibition of the tested pathogens was shown by the Lactobacillus uvarum LUHS245 and Lactobacillus casei LUHS210	(Bartkiene et al. 2019)
Sweet cherry processing by-products	Pseudomonas aeruginosa (ATCC 9027), Enterococcus faecalis (ATCC 29212), Staphylococcus aureus subsp. aureus (ATCC 6538P), Escherichia coli (ATCC 8739), and Candida albicans (ATCC 29212)	- Free phenolic fractions of stalk exhibited remarkable antimicrobial activity against selected microorganisms except for Escherichia coli	(Yüksekkaya et al. 2021)

## Effects on physical, chemical, and nutritional properties of food products by the addition of FBPs

The Valorization of FBPs can have various physical, chemical, and nutritional effects on the final product. These effects are influenced by factors such as the type of FBPs used, their processing, and the composition of the fortified food. Different processing methods can have varying effects on the physical, chemical, nutritional, and sensory attributes of the by-product and the fortified food (Larrosa and Otero 2021; Rodríguez-Solana et al. 2021). Processing can change the size and texture of FBPs. For instance, chopping, grinding, or pureeing by-products can result in smaller particle sizes, which may be more evenly distributed throughout the fortified food. This can impact the texture and mouthfeel of the finished goods. The effects of the addition of FBPs on the physical, chemical, and nutritional properties of food products are presented in Table 4 as an example. In an earlier study, Niu et al. (2014) suggested smaller particles (< 40 µm) of FBPs to develop smoother surfaces as well as increased chewiness and hardness of cooked noodles. Moreover, proper processing techniques can ensure the uniform distribution of by-products within the fortified food. This is essential to prevent clumping or uneven distribution, which can affect product consistency. Some processing methods, like drying or dehydration, can reduce the water content of FBPs. This can lead to concentrated flavors and extended shelf life but may also affect the moisture balance of the fortified food, potentially making it drier. The processing method employed can impact the retention of nutrients in the by-products. For instance, heat-based processes like pasteurization or blanching can result in some nutrient loss, particularly heat-sensitive vitamins (Mieszczakowska-Frąc et al. 2021). Freeze-drying or vacuum drying, on the other hand, may preserve better nutrient content (Harguindeguy and Fissore 2020). Some processing methods, such as evaporation, can concentrate the natural flavors of FBPs. This can intensify the fruity or tangy notes and influence the overall taste of the fortified food. However, over-concentration can lead to an overly strong or artificial flavor.

**Table 4:** Changes in physical, chemical, and nutritional properties of foods by the addition of FBPs

Fruit By-products	Targeted Product	Changes in physical properties	Changes in chemical properties	Changes in nutritional properties	References
Mango peel powder	Bread	– Bread height, specific volume, and weight loss percentage decreased – Density increased	– Starch digestibility percentage decreased	– Total phenol (mg GAE/100 gm) increased 220.33 ± 8.5 to 757.8 ± 13.5 at 5% incorporation – Antioxidant activity increased at least 3 times after 5% addition	(Chen et al. 2019; Pathak et al. 2016)
	Tortilla chips	– Lowered the required fracture force – Influenced pore formation	– Ash, fiber, and phenol content increased significantly – Glycemic index lowered	– Total phenols (mg/g) increased 0.61 ± 0.05 to 3.73 ± 0.14 at 10% incorporation – Total dietary fiber enhanced almost 3 times than control	(Mayo-Mayo et al. 2020)
	cookies	– Decreased the diameter of cookies – Breaking strength increased	– Fiber, carotenoids, and polyphenolics enhanced	– Carotenoids (mg/g) increased 17 ± 3 to 247 ± 14 at 20% incorporation – Polyphenolics enhanced almost 5 times and dietary fiber enhanced almost 3 time	(Ajila et al. 2008)
Banana peel extract	Yogurt	– Viscosity increased – Syneresis percentage slightly increased	– pH value slightly increased	– α-glucosidase inhibition percentage raised 72.11 ± 2.34 to 81.38 ± 3.20 after incorporation – Phenols and antioxidant activity doubled	(Kabir et al. 2021)
Sweet potato peel flour	Arabic bread	– Roundness of the arabic bread decreased	– Better mineral concentration – Higher crude fiber and ash content – Higher phytochemical content	– Total phenol, total flavonoid, and total anthocyanin were almost tripled at 5% incorporation	(Elkatry et al. 2023)
Banana peel powder	Chicken Sausage	– Reduced cohesiveness and increased brittleness – Water holding capacity increased	– Decreased lipid oxidation	– Dietary fiber almost doubled at 2% incorporation	(Zaini et al. 2020)
Prickly pear peel flour	Bread	– Volume decreased and weight increased – Cohesiveness and springiness increased	– Moisture content and water activity increased – Significant increase in total polyphenols	– Phenols and flavonoids activity increased almost eightfold and twofold, respectively, at 20% addition	(Parafati et al. 2020)
Grape juice residue	Ice cream		– Carbohydrate, ash, and total phenolic content increased significantly	For 2% addition: – Total dietary fiber (%) increased 0.27 ± 0.01 to 1.09 ± 0.06 – Total phenolics (GAE mg/100 g) increased from 129.40 ± 0.35 to 197.41 ± 0.18 – Flavonoids (mg CE/100 g) increased from 128.17 ± 0.68 to 184.20 ± 5.38	(Nascimento et al. 2018; Vital et al. 2018)
Carrot pomace powder	Donut	– Specific volume decreased – Crumb firmness enhanced	– Dietary fiber increased – Moisture content increased		(Nouri et al. 2017)
Pineapple peel	Vienna sausages	– Reduced the release of whey	– Dietary fiber increased – Water holding capacity increased	– Significant increment in carotenoid and antioxidant capacity	(Montalvo-González et al. 2018)
Watermelon rind powder	Cake, Noodles	– Cake volume decreased – Cake weight increased	– Moisture content increased in cake	– In noodles total phenolic content (mg GAE/kg dm) increased 82.5 ± 7.63 to 1164.0 ± 6.15 at 15% incorporation – Crude fiber enhanced almost 24-fold at 15% incorporation	(Ho and Che Dahri 2016; Hoque and Iqbal 2015)
Jackfruit seed powder	Cake	–	– Protein content enhanced – Nutritional value improved	– Protein, fiber, and ash content improved significantly at a 1:1 mixture ratio	(Sultana 2017)

The composition of fortified food, including the choice and quantity of ingredients, can have a considerable impact on the end product. The composition determines the nutritional profile, taste, texture, stability, and overall sensory characteristics of the fortified food. The primary purpose of fortification is to enhance the nutritional content of the food. The type and amount of fortificants (e.g., antioxidants, vitamins, minerals, fiber) added will directly influence the nutritional profile of the final product. In an earlier study, Kabir et al. (2021) reported that the phenol content of fortified yogurts increased along with increased concentration of banana peel extract added to it. Thus, it's crucial to achieve the desired nutrient levels without over-fortification, which can lead to health risks or undesirable taste and texture changes. The interaction between fortified nutrients and other components in the food can affect nutrient bioavailability. For example, the presence of certain minerals may interfere with the absorption of others. Formulating to optimize nutrient synergy and minimize antagonism is essential (Henry and Heppell 2002).

The types, processing of the FBPs and the composition of food plays a crucial role in determining how they will affect the fortified food. Manufacturers must carefully select the FBPs, their processing methods based on the desired features of the finished product. Balancing factors such as nutrient retention, flavor concentration, and texture modification is essential to create a fortified food that meets both nutritional and sensory goals. Additionally, quality control measures during processing are critical to ensure consistency and safety in the fortified food production process.

## Effects on sensory attributes of food products by the addition of FBPs

The quality and safety of a food item can greatly improve through the incorporation of FBPs depending on the selection of the best-added doses. However, one should consider the sensory modification (e.g., regarding color, flavor, taste, mouth-feel, texture, and overall acceptability descriptors) in addition to FBPs. For instance, aromas can be affected by the presence of monoterpenes (limonene, mircene), sesquiterpenoids (α and β-sinensal), and sesquiterpene (valencene) found in FBPs (Teixeira et al. 2020). Carotenoids especially β–carotene as pigments present in FBPs, can be used to enhance the color and appearance of finished foods and beverages (Christaki et al. 2013). The effect of the addition of FBPs in food products sensory attributed is presented in Table 5 as an example. It has been reported that the color changes by the addition of valuable ingredients from FBPs into food might reduce consumer acceptance. Thereby, the formulation that improves or at least holds the sensory attributes of the original item must choose by one for FBPs fortification (Trigo et al. 2020). Accordingly, one must select the formulation, which improves or at least hold the original characteristics of the products.

**Table 5:** Sensory effect of foods by the addition of FBPs

FBPs	Food products	Target issue	Effects	References
Banana peel	Yogurt	Phenolic compounds	- Increase total phenolic content, antioxidant activity - Reduce lipid oxidation and increase viscosity - Good sensory acceptability	(Kabir et al. 2021)
Mango by-product	Bakery products, biscuits	Dietary fiber and polyphenols	- Increase in fiber and polyphenol content - Increase in weight, density, and breaking strength - Good sensory acceptability	(Martins et al. 2017)
Apple pomace	Wheat bread	Nutritional value and physicochemical, antioxidant, and sensory properties	- Increase in ash and total carbohydrate content - Increase of total polyphenol content and antioxidant activity - No substantial alteration in sensory attributes	(Valková et al. 2022)
Banana peel	Bakery products, Pasta, Confectionaries	Ash, dietary fiber, and total phenolic content	- Increase of fiber, ash, and phenolic content - Increase of gumminess, volume, viscosity, and foaming stability - Increase overall sensory acceptability	(Martins et al. 2017)
Watermelon rinds	Cookies	Dietary fiber and bioactive compounds	- Increase in dietary fiber and antioxidant content - Reduce glycemic index - Products with slightly softer texture - Good sensory acceptability	(Naknaen et al. 2016)
Pineapple Peel	Yogurt	Dietary fiber	- Increase in dietary fiber - Reduce firmness and fermentation time	(Sah et al. 2016)
Orange peel	Orange jam	Sensory, physicochemical, and Nutritional value	- Increase of sugars, soluble solids, titratable acidity, and water activity - Enhanced levels of antioxidants, carotenoids, phenolic compounds, protein, ash, lipids, and fiber - Jam with 8% orange peel maintains sensory acceptability	(Teixeira et al. 2020)
Kinnow rind, pomegranate rind, and seed powders	Goat meat patties	Antioxidant compounds	- Increase in free radical-scavenging phenolic compounds - Reduced lipid oxidation - No significant change in sensory attributes	(Devatkal et al. 2010)
Lemon, orange, grape, and banana fruit peel powders	Chicken patties	Oxidative stability, microbial quality, physicochemical properties, and sensory attributes	- Increase bioactive substances and free radical scavenging activity - Significant antioxidant and antibacterial activities - Improved sensory attributes	(Abdel-Naeem et al. 2022)

## Challenges and barriers to the use of FBPs for the development of innovative healthy foods

The existence of valuable compounds in fruit waste has been recognized for a long time, but the prospect of those compounds is beginning to emerge recently. Many more aspects of utilizing those valuable substances are unknown to us. Adequate research and knowledge are required to establish and implement this idea. For example, there are a number of works demonstrated the existence of valuable compounds in food waste (Mutua et al. 2016; Mugwagwa and Chimphango 2019; Islam et al. 2021). Many of the studies even have enriched foods with the help of those exquisite compounds and showed dramatic changes in the nutritional component of those foods (Ajila et al. 2008; Kabir et al. 2021; Panza et al. 2021). But very few of them studied the long-term effects of taking those enriched foods. There is not enough data towards the synergistic effect of recorded dietary habits. Moreover, specific bioactive compounds or their combination may not be a good fit for a particular chronic condition or age. So, this approach of delivering bioactive compounds could be a threat to the consumer. Another great risk of utilizing FBPs is the frequent use of pesticides, insecticides, fungicides, and fertilizers for better fruit production. Those are often used on fruits by cultivars to prevent unwanted fruit spoilage by insects, pests, rodents, bacteria, fungus, and in some cases viruses (Hassaan and Nemr 2020). Toxins formed by biochemical processes, such as mycotoxins, aflatoxins, and fumonisins may represent a concern for the safety of food because of their ubiquity and a range of detrimental effects on human health (Kumar et al. 2022). These toxins may produce in the by-products and that can damage human health if consumed. Fruit by-products may contain anti-nutritional elements (Such as saponins, alkaloids, tannins, phytates, oxalates) and heavy metals (such as lead, chromium, cadmium, etc.) that could have a negative influence on the metabolic processes of the human body. Moreover, fruits that are exported are subjected to a protracted handling and supply chain, which frequently results in quality loss such as rotting, shriveling, over-ripening, and weight loss. To maximize the economic feasibility of its goods, the fruit and vegetable business heavily relies on the post-harvest use of synthetic chemicals including chlorine dioxide, nitric oxide, 1-methyl cyclopropane, and salicylic acid (Choi et al. 2016; Kumar et al. 2017). These harmful elements outcast human health in vulnerable conditions. According to a study conducted by World Health Organization (WHO), the improper use of pesticides is directly causing acute poisoning in around 1 million people every year where the death rate is 0.4–1.9% (Zhang et al. 2020). So, before utilizing the FBPs in a big margin, adequate knowledge and research are required to acquire the proper information on the mentioned concerns.

The incessant need for fruit’s bioactive compound drives the quest for efficient, non-thermal, and GRAS (generally regarded as safe) status for extraction techniques. The appropriate extraction technique depends on the types of by-products and the sort of bioactive compounds to be isolated. For example, the best technique for carotenoid extraction is supercritical fluid extraction, while polyphenols are more sustainable in pressurized solvent extraction (Garcia-Mendoza et al. 2015). Therefore, to separate the specific bioactive components for commercial application, it needs the right extraction facilities, which might result in higher costs for the enterprises. Consumer acceptance is another issue for accepting fruit waste and by-products as a source of bioactive compounds. The sensory alteration such as a change in color, off-taste/odor, textural change, odd mouth-feel, and overall acceptability may be a key factor for utilizing those valuable components. However, in practice, people choose a product formulation that enhances or at the least preserves the product's original sensory qualities. It has been reported that the use of FBPs often significantly alters the sensory characteristics of products. In a study, it is demonstrated that passion fruit peel and seed flour darken the color of yogurt and increase its viscosity after incorporation (de Toledo et al. 2018). Thereby, maintaining the sensory qualities of fortified or enriched products may be a big challenge for the utilization of bioactive compounds derived from FBPs.

## Solutions for increasing the usage of FBPs

Bioactive compounds from FBPs have been incorporated into human food for value addition for quite a few decades. Phytochemicals, including phenolics, anthocyanins, and carotenoids are naturally occurring functional components that have a variety of health-improving properties, such as antioxidant, anti-diabetic, and anti-cancer properties (Zhang and Watson 2004). In addition to their health-promoting effects, fruit waste's phytochemicals include antibacterial and antioxidant properties that help food products last longer by postponing or preventing lipid oxidation (Kabir et al. 2021) and the development of food-borne microbes (Ismail et al. 2016).

Many researchers have introduced different solutions by formulating various food products considering the benefits of FBPs through innovative technologies such as extrusion, encapsulation, and 3D food printing. These technologies have opened up exciting opportunities for developing functional foods by the utilization of FBPs that were previously discarded or underutilized, thereby reducing waste and promoting sustainability. Here's a summary of how these technologies are applied:

Extrusion is a versatile technology used to process fruit by-products into functional food ingredients. This process involves mixing the by-products with other ingredients (such as starches, flavorings, and binders) to form a dough-like consistency. This dough is then extruded through a specially designed extruder to create various shapes and sizes by the application of heat, pressure, and shear to a food mixture (Alam et al. 2016). These snacks can be dried or baked to remove moisture, resulting in a crunchy and flavorful product. This versatile process can accommodate a wide range of ingredients, making it ideal for handling fruit by-products. For example, functional noodles were developed through this technology by the inclusion of powdered mango fruit peel to enhance bioactive compounds and functional activity (Kabir et al. 2024 ). Researchers found that some changes may occur during the extrusion process which may be simple or complex and can encompass protein denaturation, polymerization, solubilization, gelatinization, denaturation of vitamins, browning reaction, deactivation of enzymes, and inactivation of anti-nutritional factors (Offiah et al. 2019).

Encapsulation of FBPs is a process that involves enclosing or trapping the bioactive compounds, flavors, or other valuable components found in FBPs within a protective shell or matrix (Pateiro et al. 2021). This encapsulation technique has several benefits including preservation of nutrients, controlled release, masking unpleasant tastes or odors, improved solubility, and reduced environmental impact (Dahiya et al. 2023). Encapsulated compounds can then be added to a wide range of food products, like beverages, baked goods, or supplements, to enhance their health benefits. There are various methods for encapsulating FBPs including spray drying, coacervation, extrusion, freeze drying, and emulsification (Larrea et al. 2022). The selection of encapsulation method depends on the specific application and the properties of the materials being encapsulated. Encapsulated FBPs can find applications in the food industry, pharmaceuticals, cosmetics, and more, offering a wide range of possibilities for utilizing these often-underutilized resources.

The innovative technology of 3D food printing has opened up new possibilities for reducing food waste and creating sustainable food products. The traditional method of producing food entails more unit processes and uses more energy and materials. Novel processing technologies now put an emphasis on the environment's effect and process sustainability in addition to reducing energy, time, and prices (Misra et al. 2017). Food industries are now focusing on zero wastage practices using by-products to produce value-added foods, ensuring a balance between consumer acceptance and waste utilization. The adverse impact on the organoleptic qualities of foods caused by the use of waste items as food additives is a significant concern. To balance by-product utilization and consumer acceptance, 3D food printing technology is being used to develop novel food products. Some foods are natively printable with the addition of FBPs, while others require post-processing (Zhu et al. 2023). For example, food industry potato peel waste was valorized using 3D food printing for the preparation of noodles (Muthurajan et al. 2021). This additive manufacturing approach is anticipated to revolutionize the food industry by transforming multi-stage processes into single-stage ones (Khoo et al. 2015). 3D food printing offers an exciting opportunity to transform FBPs into valuable and sustainable food items. By reducing food waste, retaining nutrients, and promoting customization, this technology contributes to both environmental sustainability and culinary innovation, offering a promising avenue for future food production. Therefore, innovative technologies like extrusion, encapsulation, fortification, enrichment, and high-pressure processing offer various avenues for creating functional foods from FBPs. These methods not only reduce food waste but also support to the development of healthier and more sustainable food options that meet the evolving consumer preferences for nutritious and value-added products.

A discussion of the changes in bioactivities, and other prominent metrics following the incorporation of FBP into food products is described in the subsection below.

### Application in bakery products

Bioactive ingredients from FBPs can be added directly or as extract form into food products of various types. Bakery items are widely consumed on a global scale (Collar 2015). In 2017, the bread and bakery business in Europe accounted for a total consumption volume of around 30,000,000,000 kg (Popescu et al. 2021). Additionally, it is anticipated that this industry's income will increase by 2.3 percent yearly (Collar 2015). White wheat flour is typically the primary component in baked goods. Even though it is a potential source of minerals and energy, it has modest antioxidant activity because the majority of bioactive chemicals are found in the aleurone layer and bran (Dziki et al. 2014). Therefore, there are several studies regarding the fortification of bakery items using fruit by-product ingredients as shown in Tables 5 and 6.

**Table 6:** Application of FBPs directly to food items fortification

Fruit Source	By-products	Fortified functional compound	Fortified food product	Best Dose	Bioactivity	Consumer acceptance	References
Grape	Pomace powder	Carotenoids	Bread	6%	↑ FRAP, DPPH	↑ Odor (crumb, sour, and sweet), taste (sweet, sour, bitter), flavor intensity, aftertaste, off taste; = salty taste, yeast flavor	(Šporin et al. 2018)
	Pomace skin	Dietary fiber	Muffin	5–10%	= Colour, flavor, taste, texture, OA	= Colour, flavor, taste, texture, OA	(Bender et al. 2017)
	Pomace powder	Carotenoids	Cheese	5–10%	↓ Lipid oxidation (TBARS, FFA) ↓ Microbial growth	↑ Flavor, texture, OA; = appearance, sourness	(Costa et al. 2018)
	Pomace skin	Phenolics compounds	Yoghurt	6%	↑ DPPH = Viability of the starter culture	–	(Costa et al. 2018)
Pomegranate	Peel powder	Phenolics compounds, dietary fiber	Cookie	1.5–7.5%	↑ FRAP, DPPH ↓ Microbial growth ↓ Lipid oxidation (TBARS)	↓ Taste, color, crispness, texture, OA	(Ismail et al. 2016)
	Bagasse	Phenolics compounds, dietary fiber	Bread	15%	↑ DPPH	↑ Flavor; ↓ appearance, texture, color, taste, mouth feel, OA	(Bhol et al. 2016)
Orange	Peel powder	Dietary fiber	Cake	12.5%	–	= Appearance, color, odor; ↓ flavor, texture	(Bhol et al. 2016)
Apple	Endocarp powder	Dietary fiber	Cookie	15%	–	↑ Taste; = appearance, aroma, texture, OA	(de Toledo et al. 2017)
	Pomace powder	TPC, TFC, TDF	Biscuit	9%	↑ DPPH, reducing power	↑ Flavor; = OA; ↓ appearance, color, and texture;	(Mir et al. 2017)
	Pomace		Milk-based desert	0.5–3%	↑ Survival rates of probiotic bacterium ↑ Microbial growth	↓ Flavor, texture, OA; = appearance, mouthfeel	(Ayar et al. 2018)
Mango	Peel powder	TPC	Bread	3%	↑ FRAP, DPPH	↑ Fruity aroma, fruity taste, aftertaste, crumb color, hardness, stickiness; ↓ bread aroma	(Pathak, et al. 2016)
Pineapple	Peel powder	TDF	Yoghurt	1%	↓ Fermentation time	–	(Sah et al. 2016)
	Central axis powder	TDF	Cookie	15%	–	↑ Taste; = appearance, aroma, texture, OA	(de Toledo et al. 2017)
Passion fruit	Peel powder	TDF	Bread	20%	–	↑ Difference intensity; = appearance, aroma, texture, flavor, OA	(Conti-Silva and Ferreira Roncari 2015)

In bread fortification, a portion of wheat flour was substituted with grape pomace powder at varying concentrations. Adding 6% grape pomace induced the best overall quality of the bread, the antioxidant activity of fortified bread was increased, and the inclusion of grape pomace flour resulted in a stickier and less bouncy crumb (Šporin et al. 2018). Quiles et al. (2018) fortified bakery products with FBPs to reduce calorie content and enhance antioxidant activity, and soluble dietary fiber. Mir et al. (2017) fortified crackers with 9% apple pomace powder and found higher dietary fiber contents than the control. Oliveira et al. (2016) studied the partial replacement of wheat flour in cakes with orange peel powder in different formulations. Doses with 12% orange peel powder showed a significant increase in dietary fibers. Several researchers also found similar results in the fortification of bakery products in different formulations with FBPs (Bhol et al. 2016; Conti-Silva and Ferreira Roncari 2015; Pathak et al. 2016).

The form of the by-product can significantly affect the sensory properties, physicochemical, microbiological, and of a fortified product. Ismail et al. (2016) used both pomegranate peel powder and extract in wheat flour cookies. The amount of total dietary fiber greatly increased when powdered peel in place of wheat flour was used; however, peel extract did not have the same effect. In contrast, when compared to the maximum dose of powdered peel, all extract concentrations demonstrated larger TPC and antioxidant potential values. Throughout storage, the microbiological evolution followed a similar pattern, as total plate counts were on average 45 percent lower in cookies supplemented with peel extract. Ben Jeddou et al. (2017), in their study of introducing potato peel powder to a cake formulation, produced equivalent outcomes. The sensory factors tested in this instance were crumb color, aroma, flavor, tenderness, appearance, and overall acceptance. Even while there were no statistically significant changes between the control cakes and tested concentrations (2–10 g/100 g), it was evident that increasing replacement doses diminished most sensory properties (Ben Jeddou et al. 2017).

### Application in dairy products

Dairy foods contain high-fat contents and are susceptible to lipid oxidation. Bioactive compounds from FBPs are used to retard the oxidation process, some examples are shown in Tables 6 and 7. Costa et al. (2018) fortified cheese with grape pomace and the results showed that bioactive compounds of grape pomace significantly decreased lipid oxidation and microbial growth. There have been many studies done to improve the phenolic compounds of the milk products (Costa et al. 2018; Sandhya et al. 2018). Demirkol and Tarakci, (2018) have found that yogurt fortified with grape peel pomace can significantly increase phenolic compound and antioxidant activity.

**Table 7:** Fortification of food products with the extract of FBPs

Fruit source	By-products	Fortified functional compound	Fortified food product	Best Dose	Bioactivity	Consumer acceptance	References
Banana	Peel extract	-	Orange juice	500 mg/100 mL	↑ ABTS, FRAP = Lipid oxidation (β-carotene bleaching)	↑ Flavor = color	(Ortiz et al. 2017)
Pomegranate	Peel extract	↑ TPC = TDF	Cookie	0.3–1.0 g/100 g flour	↑ FRAP, DPPH ↓ Microbial growth ↓ Lipid oxidation (TBARS, FFA)	↓ Taste, color, crispness, texture, OA	(Ismail et al. 2016)
	Peel extract	↑ TPC	Apple juice	500 mg/100 mL	↑ Reduction of Fremy's salt radical; = ABTS	= Colour, odor, sweetness, acidity, flavor, OA	(Altunkaya et al. 2013)
	Peel extract	TPC	Dealcoholized wine	160 mg/100 ml	-	↑ Acidity, astringency, yeast odor, and flavor; ↓ berry and apple flavor; = wine and berry odor	(Kulichová et al. 2018)
	Peel extract	↑ TPC	Curd	1%	↑ ABTS, DPPH ↓ Microbial growth	↑ Flavor, texture, OA; ↓ color	(Sandhya et al. 2018)
Grape	Pomace extract	↑ TPC, catechin and epicatechin content	Apple juice	50 mg/100 mL	↑ ABTS, FRAP	= OA	(Kulichová et al. 2018)
	Press oil cake extract	↑ TPC, catechin and epicatechin content	Grape juice	50 mg/100 mL	↑ ABTS, FRAP	= OA	(Kulichová et al. 2018)
Orange	Peel extract	TPC	Fermented milk	1 g/ 100 ml	= Growth of probiotic bacteria	–	(Casarotti et al. 2018)
	Peel extract	TPC	Yoghurt	600 mg/100 g milk	↑ Growth of starter culture during fermentation	↑ Cohesiveness, adhesiveness, taste, whey exudation; ↓ aftertaste	(Arioui et al. 2017)

FBPs can have either good or unwanted effects on the sensory characteristics of the finished product; it depends on the (i) dose, (ii) form of the by-product (usually powder or extract), and (iii) dairy matrix of the fortified product. After 15 days of storage, pomegranate peel extract considerably enhanced the flavor, texture, and acceptability of curd (1000 mg/100 mL) vs. control (Sandhya et al. 2018). Additionally, the cohesion, adhesiveness, and flavor descriptors in a yogurt matrix enhanced with orange peel extract (600 mg/100 g milk) performed better in comparison to control samples, although the aftertaste was adversely impacted (Arioui et al. 2017).

### Application in beverage

Fruit beverages are popular drinks all over the world. The global fruit juice market value was 141 billion USD in 2021. Many studies are working on the utilization of FBPs by fortification of beverages (Tables 6 and 7). The majority of research showed that adding FBPs improved the TPC and/or antioxidant activity of beverages. For example, antioxidant activity for fresh orange juice fortified with banana peel extract (500 mg/100 mL) increased by roughly 21 and 150 percent, respectively, in comparison to the non-fortified juice. Altunkaya et al. (2013) observed in their study when pomegranate peel extract (500 mg/100 mL) was added to apple juice, the relative level of free radicals dropped by 5% while TPC was amplified by 19% in contrast to the control. Kulichová et al. (2018) have found that TPC, FRAP, DPPH, catechin, and epicatechin concentrations improved by roughly 70, 140, 190, 50, and 160 percent, compared to non-fortified juices when an extract of grape press oil cake was added (50 mg/100 mL). The same extract was employed by the authors to fortify apple juice and got comparable outcomes using the procedures mentioned above. Pomegranate peel extract (160 mg/100 mL) was added to dealcoholized red wine in another study to produce a beverage with a stronger yeasty flavor, aroma, and acidity. It was also evident that the intensity of berry and apple flavor decreased, where there was no effect on wine and berry flavor compared to the control (Tárrega et al. 2014).

## Nutraceutical potential and health benefits of FBPs

Cardiovascular diseases are the primary cause of death worldwide among conditions with significant rates of morbidity and mortality in the population. Certain factors such as obesity, high blood sugar, high blood pressure, high cholesterol, smoking, eating habits, and lack of physical exercise are associated with cardiovascular disease (Farinazzi-Machado 2017). These factors can be significantly maintained by introducing FBPs into our foods. There has been in-vivo and in-vitro research going on the antioxidant, antihypertensive, and anti-inflammatory properties of FBPs as shown in Table 8. Camu-camu (*Myrciaria dubia*) seeds contain an abundance of phenolic compounds including gallic and ellagic acids, cyanidin-glucoside, malvidin-diglucoside, and quercetin rutinoside (Fidelis et al. 2020). These chemicals decrease oxidation reactions by 24 to 86% in male Wistar rat egg homogenates, with the greatest inhibition observed in water and ethyl alcohol extracts. As in the scenario of yellow fruits, peel powder is a by-product of the juice factory that contains significant levels of antioxidants and nutritional fiber, both of which can contribute to minimize cardiovascular variables. In this context, Vuolo et al. (2020) assessed the effects of fruit by-products on weight increase, obesity, oxidative damage, and proinflammatory cytokines; their findings revealed that the inclusion of dietary fiber (both soluble and insoluble) might reduce lipid uptake, lipid oxidation levels, fat formation, and antioxidant defense system.

**Table 8:** Bioactive compounds and their health benefits from FBPs

Fruit Source	By-products	Biological activity	Effect	References
Camu-Camu	Seed extract	Cardioprotective	Inhibition activity of ACE-I and lipid peroxidation	(Fidelis et al. 2020)
Passion fruit	Peel and seed extract	Cardioprotective	ACE-I activity inhibition	(Vuolo et al. 2020)
Mango	Peel and seed kernel extract	Anti-diabetic	Inhibition of α-amylase and α-glucosidase activity	(Jonatas et al. 2020)
Pomegranate	Peel extract	Anti-diabetic	Reduced fasting blood glucose levels, renal toxicity indices, and body weight restoration	(Manna et al. 2019)
Lychee	Seed extract	Neurological protection	Stabilization of intracellular microtubules and neuroprotection	(Savla et al. 2017)
Avocado	Peel extract	Neurological protection	Increased life expectancy and locomotor activity, as well as a reduction in lipid peroxidation	(Ortega-Arellano et al. 2019)
Pineapple	Peel, stalk, and stems extract	Anti-cancer	Growth inhibitor for MCF7 breast cancer cells	(Haiyan et al. 2020)
Araticum	Peel and seed extract	Anti-cancer	Cytostatic action	(Prado et al. 2020)

One of today's largest health crises is diabetes. In 2015, it was predicted that almost 400 million individuals globally have diabetes mellitus, and by the year 2035, that number is expected to rise to 471 million (Adefegha et al. 2015). The most prevalent kind of diabetes is type 2 which is due to rapid societal and lifestyle changes, a decrease in physical activity, and a diet poor in fruits and vegetables, among others (Cádiz-Gurrea et al. 2020). In this regard, several efficient anti-diabetic medications come in a variety of forms with various modes of action, but they are all linked to potential negative side effects. As a result, research on phytochemicals that may serve as possible adjuvant medicines was conducted in the past year. Since diabetes is linked to oxidative stress, antioxidant chemicals from FBPs will emerge as a more enticing substitute (Ishak et al. 2019). Many authors studied the antidiabetic activity in FBPs. Jonatas et al. (2020) have used mango seed extract to get bioactive compounds that lowered fasting blood sugar levels by a lot in rats made diabetic by alloxan. Also, pomegranate peel extract was tested on adult male BALB/c mice that had been given a single intraperitoneal injection of streptozotocin (200 mg/kg body weight) to see if it could help diabetic nephropathy, which is caused by high blood sugar levels (Manna et al. 2019).

After cardiovascular disease, cancer is the second leading cause of death on a global scale; therefore, fresh alternative medicines are necessary for the diagnosis and treatment of this illness. FBPs are a rich source of naturally occurring anticancer chemicals (Mirza et al. 2021). Bromelains derived from pineapple peel, pulp, young and mature stems, and fruit stalk have been investigated for their potential to prevent the proliferation of MCF7 breast cancer cells, considering that extracts from the peel and pulp had the highest survival rate (Haiyan et al. 2020). Accordingly, eight different human cancer cell lines were subjected to tests with phenolic compounds derived from araticum peel and seed extracts: prostate (PC-3), multidrug-resistant ovary carcinoma (NCIADR/RES), glioma (U251),), ovary (OVCAR-03), breast (MCF-7), leukemia (K562), and non-small cell lung cancer (NCI-H460 colon (HT-29) (Prado et al. 2020). All these seed and peel extracts had an antitumor activity on examined tumor cell lines, whereas seed extract had the highest total percentage inhibition values.

Neurological disorders are becoming more and more recognized among the most common types of illness in the world. Parkinson's and Alzheimer's are two of the most prevalent neurodegenerative diseases that become much more common in old age people. Both disorders have neuropathological signs that are caused by various mechanisms that are not yet completely understood. But it is generally agreed that oxidative stress is a crucial factor in the development of degenerative illnesses. This is because the brain needs a lot of oxygen and has a weak defense against oxidative damage because it doesn't have enough antioxidants. Therefore, phytochemicals like polyphenols that can get through the blood–brain barrier and get rid of high levels of harmful reactive nitrogen species (RNS) and reactive oxygen species (ROS) seem to protect in opposition to neuronal damage and against oxidative stress. Saponins from lychee seeds also stopped apoptosis caused by regulating the expression of pro-apoptosis proteins like Bcl-2 and Bax. They also stopped the NF-KB signaling pathway-associated apoptotic gene (p65) from being made and moved into the nucleus in PC12 cells (Rodriguez-Oroz et al. 2009). Parkinson's disease is characterized by a reduction in dopamine neurons in the substantia nigra (Sveinbjornsdottir 2016). Due to its high antioxidant capacity and total phenolic content, Colinred var. peels were chosen from a variety of avocado by-products from different cultivars to test the neuroprotective effects on the parkin Drosophila melanogaster model (Ortega-Arellano et al. 2019). Movement disorders caused by oxidative stress-driven neurodegeneration in the brain were brought on by the chemical paraquat, which kills dopaminergic neurons in particular. Paraquat-treated transgenic Parkin Drosophila melanogaster exhibited an increase in life duration, locomotor activity, and a reduction in lipid peroxidation when avocado peel extracts from the Colinred cultivar were administered. These benefits were primarily related to epicatechin’s antioxidant activity, with smaller contributions from catechin, B-type procyanidins, chlorogenic, and neochlorogenic acids.

## Future perspectives and final remarks

Fruit-based by-products contained a substantial number of value-added chemicals, like carotenoids and phenolics, which successively are accountable for the antioxidant ability and antimicrobial activity against bacteria involved in food spoilage processes and foodborne diseases. These functional activities could justify why FBPs are selected to be incorporated into food products. In relation to the sensory evaluation of foods fortified with FBPs, the outcomes heavily rely on factors even as the dosage, kind of by-product, and matrix within the FBPs are added. Additionally, the presence of value-added chemicals derived from FBPs in foods could improve consumers’ acceptability. For instance, it is crucial to choose the type of FBPs, their necessary pretreatments, the desired value-added constituents, the extraction method, and the parameters. Furthermore, some concerns and problems like difficulty in the application and formulation of FBPs as natural additives in foods within the food industry can be solved by the appliance of other processes like drying, encapsulation, and micro-emulsion, etc. on extracted value-added ingredients. Thus, the solutions outlined in this review paper permit an increase in the sustainable usage of FBPs, which helps extend the valorization processes additionally as protect the environment. Nevertheless, the majority of the studies under consideration do not evaluate the bioavailability and pollutants of FBPs, which are necessary to determine whether these innovative products are safe and healthy for consumers.

Nowaday’s FBPs management and utilization are a big concern worldwide. Although safety concerns are becoming more prevalent in this industry, new procedures and approaches are being created and used to lessen their impact and increase their value. Safety evaluation investigations, such as microbiological study, physicochemical quality, and/or contaminant determination were only included in a small fraction of published papers. Constant effort would be needed to educate both consumers and food entrepreneurs to achieve sustainable and safe food manufacturing operations. Being open to new ideas and quick to respond to new developments is also critically important for regulatory bodies, policy-makers, and legislators. While working with closed loops of resources in the food industry, there may be several barriers to valorizing food processing by-products that may offer improved food security and new sources of sustainable health-improving bioactive compounds. Potential advancements in suitable approaches that permit the selective and sensitive determination of any hazardous material, as well as effective processes that offer enough processing without endangering the safety of the finished items should be made. In most situations, the evaluation of findings has been extremely complex and difficult due to the lack of particular regulation that controls the suitability and safety of new consumer items. The absence of this regulation puts the prospect of valorization of many potentially useful by-products from the agro-food industry in danger. This issue brings about the need for determining specific regulations for food by-product safety and valorization.

## Data Availability

Data will be available on request.
